# PD-(L)1 Inhibitors in Combination with Chemotherapy as First-Line Treatment for Non-Small-Cell Lung Cancer: A Pairwise Meta-Analysis

**DOI:** 10.3390/jcm9072093

**Published:** 2020-07-03

**Authors:** Jorge García-González, Juan Ruiz-Bañobre, Francisco J. Afonso-Afonso, Margarita Amenedo-Gancedo, María del Carmen Areses-Manrique, Begoña Campos-Balea, Joaquín Casal-Rubio, Natalia Fernández-Núñez, José Luis Fírvida Pérez, Martín Lázaro-Quintela, Diego Pérez Parente, Leonardo Crama, Pedro Ruiz-Gracia, Lucía Santomé-Couto, Luis León-Mateos

**Affiliations:** 1 Medical Oncology Department, University Clinical Hospital of Santiago de Compostela and Translational Medical Oncology Group (Oncomet), Health Research Institute of Santiago de Compostela (IDIS), CIBERONC, 15706 Santiago de Compostela, Spain; 2 Medical Oncology Department, Complexo Hospitalario Universitario de Ferrol, 15405 A Coruña, Spain; francisco.javier.afonso.afonso@sergas.es; 3 Medical Oncology Department, Centro Oncológico de Galicia, 15009 A Coruña, Spain; margarita.amenedo@cog.es; 4 Medical Oncology Department, Complexo Hospitalario Universitario de Ourense, 32005 Ourense, Spain; karmeleareses@hotmail.com (M.d.C.A.-M.); jlfirvidap@gmail.com (J.L.F.P.); 5 Medical Oncology Department, Hospital Universitario Lucus Augusti, 27003 Lugo, Spain; bcamposbalea@hotmail.com (B.C.-B.); fernandeznunez.ntalia@gmail.com (N.F.-N.); 6 Medical Oncology Department, Complexo Hospitalario Universitario de Vigo, 36213 Vigo, Spain; joaquin.casal.rubio@sergas.es (J.C.-R.); martin.lazaro.quintela@sergas.es (M.L.-Q.); 7 Lung Cancer Medical Department, Roche Farma S.A., 28042 Madrid, Spain; diego.perez@roche.com (D.P.P.); leonardo.crama@roche.com (L.C.); pedro.ruiz.pr1@roche.com (P.R.-G.); 8 Medical Oncology Department, Hospital POVISA, 36211 Vigo, Spain; lsantome@povisa.es

**Keywords:** non-small-cell lung cancer, immunotherapy, PD-1 inhibitors, PD-L1 inhibitors, chemotherapy, meta-analysis

## Abstract

The combination of programmed cell death-1 (PD-1)/programmed death ligand-1 (PD-L1) inhibitors with chemotherapy has emerged as a promising therapeutic option for advanced non-small-cell lung cancer (NSCLC). The aim of this meta-analysis was to evaluate the efficacy of the combined strategy in this setting. For this purpose, we performed a literature search of randomized controlled trials comparing PD-(L)1 inhibitors plus platinum-based chemotherapy versus chemotherapy alone in stage IV NSCLC patients. Seven clinical trials with 4562 patients were included. In the intention-to-treat wildtype population, PD-(L)1 inhibitor plus chemotherapy was significantly associated with improved progression-free survival (PFS) (Hazard ratio (HR) = 0.61, 95% confidence interval (CI): 0.57–0.65, *p* < 0.001) and overall survival (OS) (HR = 0.76, 95% CI: 0.67–0.86; *p* < 0.001) compared to chemotherapy. A significantly higher overall response rate (ORR) was also observed with the combined strategy (Odds ratio (OR) = 2.12, 95% CI: 1.70–2.63, *p* < 0.001). Furthermore, in all the analyzed subgroups, addition of PD-(L)1 inhibitors to chemotherapy significantly improved efficacy endpoints. Specifically, stratification according to PD-L1 expression revealed a benefit across all patients, regardless of their PFS status. In conclusion, PD-(L)1 blockade added to standard platinum-based chemotherapy significantly improved PFS, OS, and ORR in the up-front treatment of advanced NSCLC.

## 1. Introduction

Lung cancer remains the leading cause of cancer-related death worldwide among men and the second among women [1]. Non-small-cell lung cancer (NSCLC), which is the most common type, accounts for 80% to 85% of all lung cancer diagnoses [2]. It is frequently diagnosed in the advanced stage, with 5-year survival rates ranging from 0% to 5% with chemotherapy, the only systemic therapeutic strategy available for decades [3]. In this regard, blockade of the programmed cell death-1 (PD-1)/programmed death ligand-1 (PD-L1) axis in particular has opened up a new horizon in the lung cancer therapeutic landscape, increasing overall survival (OS) not only in patients with advanced NSCLC but also in patients with stage III NSCLC and extensive-stage small-cell lung cancer [4,5,6].

Since 2015, three different PD-(L)1 inhibitors have been approved by the European Medicines Agency (EMA) and/or the U.S. Food and Drug Administration (FDA) for the treatment of metastatic NSCLC (mNSCLC) [7]: two anti-PD-1 antibodies (nivolumab and pembrolizumab) and one anti-PD-L1 antibody (atezolizumab), indicated for patients regardless of their PD-L1 expression status (nivolumab and atezolizumab) or for PD-L1-positive patients only (pembrolizumab). All of them have demonstrated an improvement in OS compared to docetaxel in second-line therapy [8,9,10]. In the first-line setting, results from the KEYNOTE 024 trial demonstrated that, compared with platinum-based chemotherapy, OS, progression-free survival (PFS), and overall response rate (ORR) were significantly improved in patients with PD-L1 expression on at least 50% of tumor cells and without oncogenic driver mutations [11,12]. Interestingly, an additional study assessing pembrolizumab efficacy versus chemotherapy using a PD-L1 tumor proportion score (TPS) of 1% or greater (KEYNOTE-042 [13]) demonstrated improved OS for the full cohort, which, despite being higher for higher PD-L1 expression, supported a potential extended role of pembrolizumab monotherapy as a standard first-line treatment for PD-L1-expressing advanced/metastatic NSCLC [14]. In contrast, nivolumab did not demonstrate statistically significant survival benefits in previously untreated PD-L1-positive mNSCLC (CheckMate-026 [15]).

Nevertheless, many patients with advanced NSCLC do not benefit from PD-(L)1 inhibitors, either in the first line or in the second or successive lines of treatment. The search for reliable predictive biomarkers of response to these drugs is therefore essential to improve patient outcomes.

The potential synergistic effects of combining chemotherapy and immunotherapy to improve the antitumor activity of anti-PD-(L)1 monotherapy were initially suggested in preclinical studies [16] (Apetoh, 2015 #16) and were further demonstrated in several clinical trials [4,5,6,17,18,19,20,21,22,23,24,25]. However, although promising outcomes have been reported, several questions remain unanswered, such as the potential real benefit for all patients at the expense of increased toxicity or the possible molecular factors that could predict the benefit of this combined therapeutic strategy.

The objective of this study was to evaluate the efficacy of the combined strategy by conducting a pairwise meta-analysis (MA) of the available information on PD-(L)1 inhibitors in combination with chemotherapy in the first-line treatment of patients with advanced NSCLC.

## 2. Materials and Methods

### 2.1. Search Strategies and Study Selection

We conducted a systematic search in PubMed to identify all eligible trials from inception until 1 January 2020, with no start date limit applied. Literature search terms used were “non-small cell lung cancer” (or “NSCLC”), “chemotherapy”, “pembrolizumab”, “nivolumab”, “atezolizumab”, “durvalumab”, and all terms related to clinical trial registration (ClinicalTrials.gov, EU Clinical Trials Register, ISRCTN, and ANZCTR). An additional search of abstracts presented at the American Society of Clinical Oncology (ASCO), European Society for Medical Oncology (ESMO), American Association for Cancer Research for Medical Oncology (AACR), and World Conference on Lung Cancer (WCLC) was also performed.

### 2.2. Selection Criteria

Only phase III trials conducted in patients with advanced/metastatic stage IV NSCLC not previously treated for their metastatic disease and receiving at least one PD-(L)1 inhibitor in combination with a chemotherapeutic agent were eligible for inclusion. Efficacy outcomes regarding combinations of immunotherapy plus chemotherapy expressed as PFS or OS had to be provided. Observational studies, editorials, reviews, and commentaries were excluded.

### 2.3. Statistical Analysis

The DerSimonian–Laird random effects models for main and subgroup analyses was implemented, assessing heterogeneity of effect-size estimates from the individual studies by Cochran’s Q test and the I^2^ statistic. Additionally, MA corresponding to analysis of binary data of proportions was also performed using a DerSimonian–Laird random effects model without transformation of the proportion. A high level of heterogeneity was considered if I^2^ was greater than 50%. Due to the relatively low number of trials involved in this MA, values and significance of heterogeneity must be considered as guidance only [26]. Statistical significance was reached for *p*-values less than 0.05. Analyses were not controlled for multiplicity; no alpha was assigned to the different analyses. The nature of this study is therefore exploratory, mainly in the subgroup analysis. Hazard ratios (HRs) and 95% confidence intervals (CIs) for OS and PFS from the overall population and subgroups from each individual trial of advanced NSCLC were calculated. For dichotomous data, odds ratios (ORs) were estimated. The MA was performed using Open Meta Analyst v. 10 (Center for Evidence Synthesis in Health, Brown University). Recommendations of the Cochrane Collaboration and the Preferred Reporting Items for Systematic Reviews and Meta-Analyses (PRISMA) guidelines were followed for this MA [27].

For the ORR, different endpoints, including complete response (CR), partial response (PR), stable disease (SD), and progressive disease (PD), were alternately modeled. These sensitivity analyses along with those for OS and PFS did not quantitatively alter the results and conclusions of the main analyses.

## 3. Results

### 3.1. Studies Included in the Meta-Analysis

**Figure 1:**
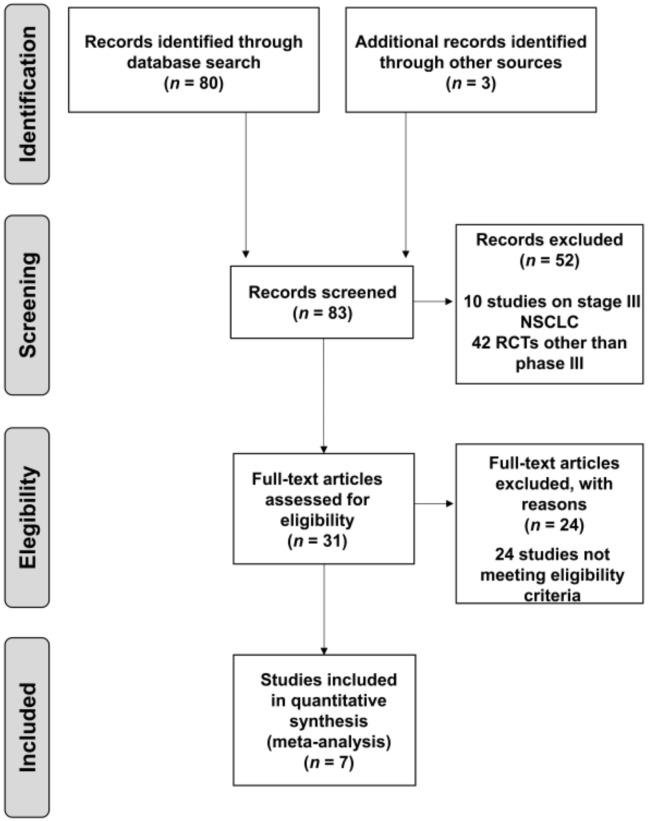
Flow chart of study selection (up to 1 January 2020). NSCLC, non-small-cell lung cancer; RCTs, randomized controlled trials.

A total of 80 records from PubMed were screened. Three additional studies presented at the WCLC and/or ESMO were also included. Study selection and exclusion criteria are summarized in Figure 1. Finally, seven clinical trials carried out with 4562 patients met the inclusion criteria and were included in the MA [17,20,21,22,23,24,25,28,29]. In the specific case of CheckMate-227, part 1 [30] was excluded because immunotherapy-plus-chemotherapy efficacy evaluation was not part of the main objectives; only part 2 was considered for this MA [21].

### 3.2. Study Characteristics

The specific characteristics of the studies included in the MA are summarized in Table 1. The control arm in all studies was platinum-based chemotherapy with pemetrexed (three studies [17,20,21,28]) or with nab-paclitaxel/paclitaxel (five studies [21,22,23,24,25,29]). In one three-arm trial (IMpower150 [24]), bevacizumab was added in the control and experimental arm. This study included two experimental arms: carboplatin, paclitaxel, and atezolizumab (arm A) and carboplatin, paclitaxel, bevacizumab, and atezolizumab (arm B) versus carboplatin, paclitaxel, and bevacizumab (arm C). No comparisons between arms A and C were performed because the HR may not reflect the actual effect of add-on immunotherapy (atezolizumab plus chemotherapy vs. bevacizumab plus chemotherapy). 

Three studies tested anti-PD1 antibodies [17,21,22,28], and four studies tested anti-PD-L1 antibodies [20,23,24,25,29]. Patients with epidermal growth factor receptor (EGFR) or anaplastic lymphoma kinase (ALK) mutations were included in two clinical trials assessing atezolizumab [24,25]. Regarding the histology, four studies included patients with nonsquamous NSCLC [20,21,24,25], two included patients with squamous NSCLC [22,29], and one evaluated patients presenting both histological types [21] (note that the primary endpoint in CheckMate-227 part 2 was OS in nonsquamous mNSCLC patients only; however, both histological types were considered for this MA).

An all-comers design was used in all the studies, with NSCLC patients entering the trial regardless of their PD-L1 expression status. Stratification was performed based on this biomarker in all trials. Thus, subjects were classified as PD-L1-negative or PD-L1-positive, and within this group, investigators distinguished patients with high or low expression levels [20,25,29]. In the atezolizumab trials [20,23,24,25,29], levels were considered high (TC3 or IC3) when PD-L1 expression was recorded on at least 50% of tumor cells or at least 10% of tumor-infiltrating immune cells (TIICs) by immunohistochemistry; levels were considered low–intermediate, or TC1/2 or IC1/2, when expression was reported on at least 1% of tumor cells or TIICs and less than 50% of tumor cells or less than 10% of TIICs by immunohistochemistry; and PD-L1-negative status, or TC0 and IC0, was determined when expression was reported on less than 1% of tumor cells and TIICs. Similar criteria were followed in trials assessing pembrolizumab or nivolumab, but PD-L1 expression was only measured in tumor cells [17,21,22,28].

According to the eligibility criteria, none of the studies included patients who had received prior treatment for metastatic disease. However, in terms of therapy for nonmetastatic disease, although most of the studies included treatment-naïve patients, in those evaluating pembrolizumab (KEYNOTE-189 [17,28] and KEYNOTE-407 [22]) or atezolizumab [20,23,24,25,29], subjects had received previous therapies (Supplementary Table S1).

Coprimary endpoints for six clinical trials were PFS and OS [17,20,22,23,24,25,28,29]. The primary endpoint for CheckMate-227 part 2 was OS in nonsquamous NSCLC patients; PFS was assessed as a secondary endpoint [21]. Mature PFS data were reported in all the studies included in this MA [17,20,21,22,23,24,25,28,29], while final data for OS were available for only one of them [21]. Interim analyses were provided for the other six studies [17,20,22,23,24,25,28,29]. Both endpoints were evaluated in the intention-to-treat (ITT) population and specifically in the wildtype population (without EGFR or ALK mutations) in the IMpower130 [25] and IMpower150 studies [23,24] (see Supplementary Table S2 for the available information on patients with mutations in IMpower150). IMpower150 was the only study in which a subsequent subgroup analysis in patients with EGFR mutations or baseline liver metastasis was performed [23]. Additionally, one or both coprimary endpoints were analyzed according to different subgroups in all the studies included in the MA (PFS in six clinical trials [17,22,23,24,25,28,29] and OS in five studies [17,21,22,25,28,29]).

Patient population characteristics of all the studies included in the MA are shown in Supplementary Table S3.

### 3.3. Efficacy Endpoints in the Overall Population

**Figure 2:**
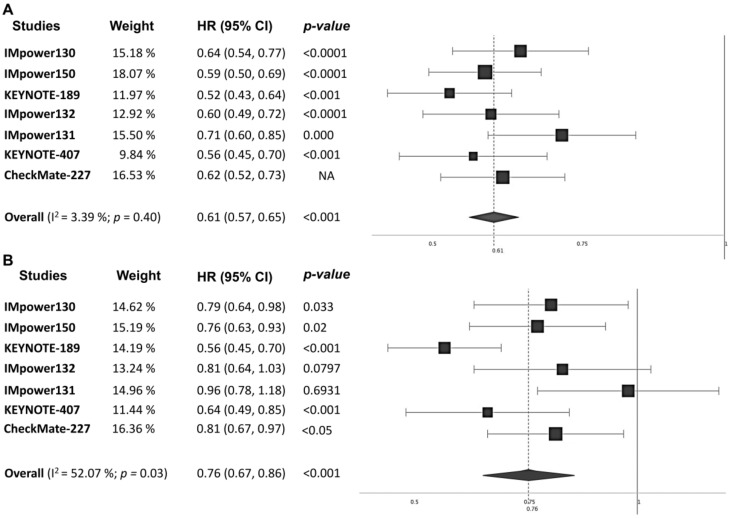
Forest plot of pooled hazard ratios for (**A**) progression-free survival (PFS) and (**B**) overall survival (OS) in patients who received programmed cell death-1 (PD-1)/programmed death ligand-1 (PD-L1) inhibitors plus chemotherapy vs. chemotherapy alone. HR, hazard ratio; CI, confidence interval.

Median PFS ranged from 4.8 to 6.8 months in the control arms and from 6.3 to 8.8 months in the treatment arms. Median OS ranged from 10.7 to 14.7 months in the control arms and from 14.2 to 22.0 months in the treatment arms. MA results demonstrated that the addition of a PD-(L)1 to chemotherapy was associated with improved PFS (PFS: HR_pooled_ = 0.61, 95% CI: 0.57–0.65, *p* < 0.001, Figure 2A) and OS (OS: HR_pooled_ = 0.76, 95% CI: 0.67–0.86; *p* < 0.001, Figure 2B) compared with chemotherapy alone. The objective response rate (ORR) was also significantly improved with the PD-(L)1 inhibitor–chemotherapy combination (odds ratio (OR_pooled_) = 2.12, 95% CI: 1.70–2.63, *p* < 0.001, Supplementary Figure S1). The best ORR values were obtained in the IMpower150 (ORR = 56.4%) trial for nonsquamous NSCLC and KEYNOTE-407 for squamous NSCLC (57.9%). Notably, in terms of both OS and ORR, there was significant heterogeneity across the six trials (I^2^ = 52.07%, *p =* 0.03; I^2^ = 67.42%, *p =* 0.005).

### 3.4. Subgroup Analysis

**Figure 3:**
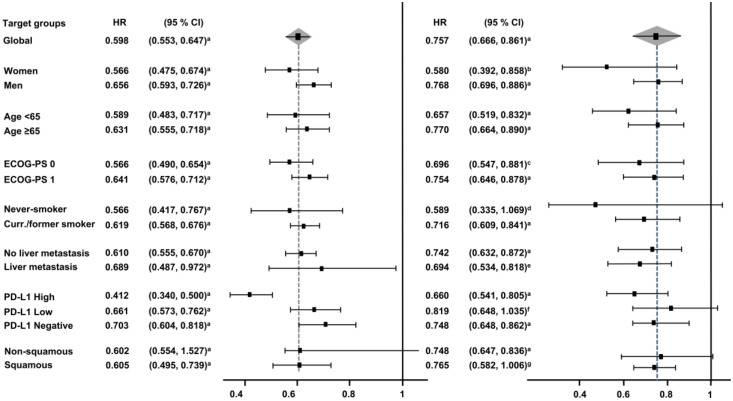
Forest plot of hazard ratios for progression-free survival (PFS) and overall survival (OS) in the subgroup analysis. PFS, progression-free survival; OS, overall survival. HR, hazard ratio; CI, confidence interval; Curr., current.; ^a^ *p* < 0.001; ^b^ *p* = 0.006; ^c^ *p* = 0.003; ^d^ *p* = 0.082; ^e^ *p* = 0.007; ^f^ *p* = 0.093; ^g^ *p* = 0.055.

Subgroup analyses according to sex (women vs. men), age (<65 years vs. ≥65 years), Eastern Cooperative Oncology Group performance status (ECOG-PS = 0 vs. ECOG-PS = 1), smoking status (never-smoker vs. current/former smoker), liver metastasis (yes vs. no), and PD-L1 expression (high vs. low vs. negative) were carried out. As shown in Figure 3, overall, the addition of PD-(L)1 blockade to chemotherapy significantly improved PFS in all the subgroups. Specifically, stratification according to PD-L1 expression revealed a benefit across all PD-L1 strata with a strong reduction in the risk of disease progression in those patients showing high expression levels (HR_pooled_ = 0.412, 95% CI: 0.34–0.5, *p* < 0.001). In terms of OS (Figure 3), although almost all subgroups benefited from the use of the PD-(L)1 inhibitor–chemotherapy combination, in certain cases, such as in never-smokers and PD-L1-low patients, results did not achieve statistical significance (HR_pooled_ = 0.589, 95% CI: 0.335–1.069, *p* = 0.082; HR_pooled_ = 0.819, 95% CI: 0.648–1.035, *p* = 0.093, respectively).

**Figure 4:**
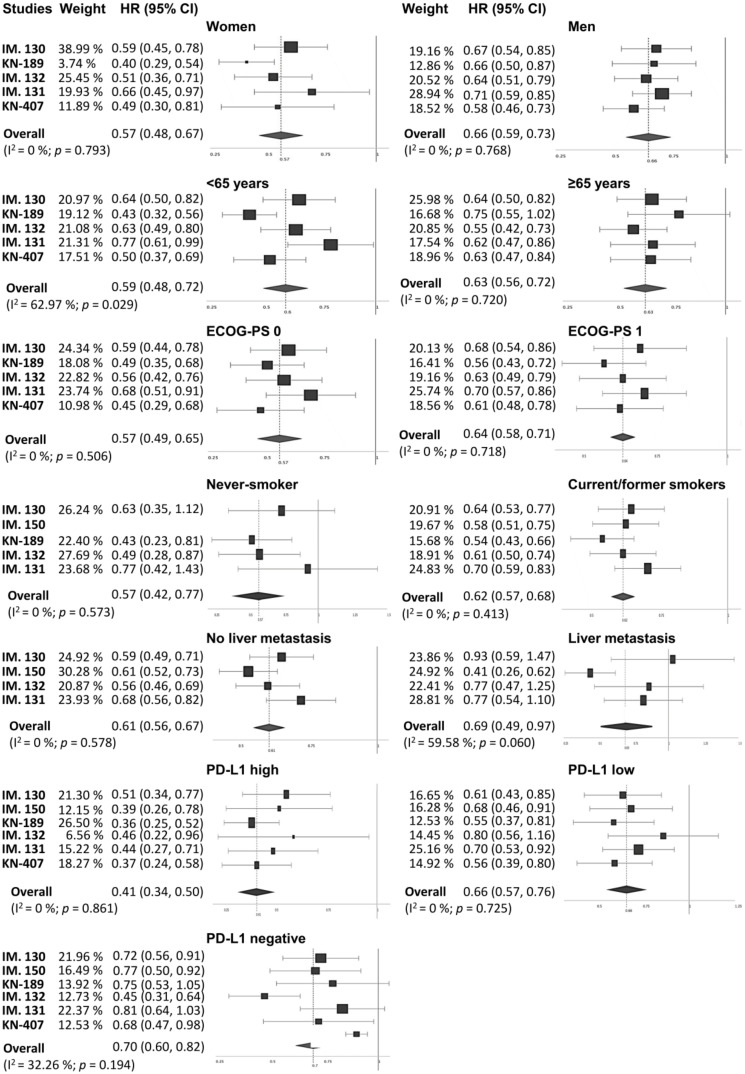
Forest plot of hazard ratios for progression-free survival (PFS) in the different patient subgroups. CI, confidence interval; ECOG-PS, Eastern Cooperative Oncology Group performance status; HR, hazard ratio; IM., IMpower; KN, KEYNOTE.

**Figure 5:**
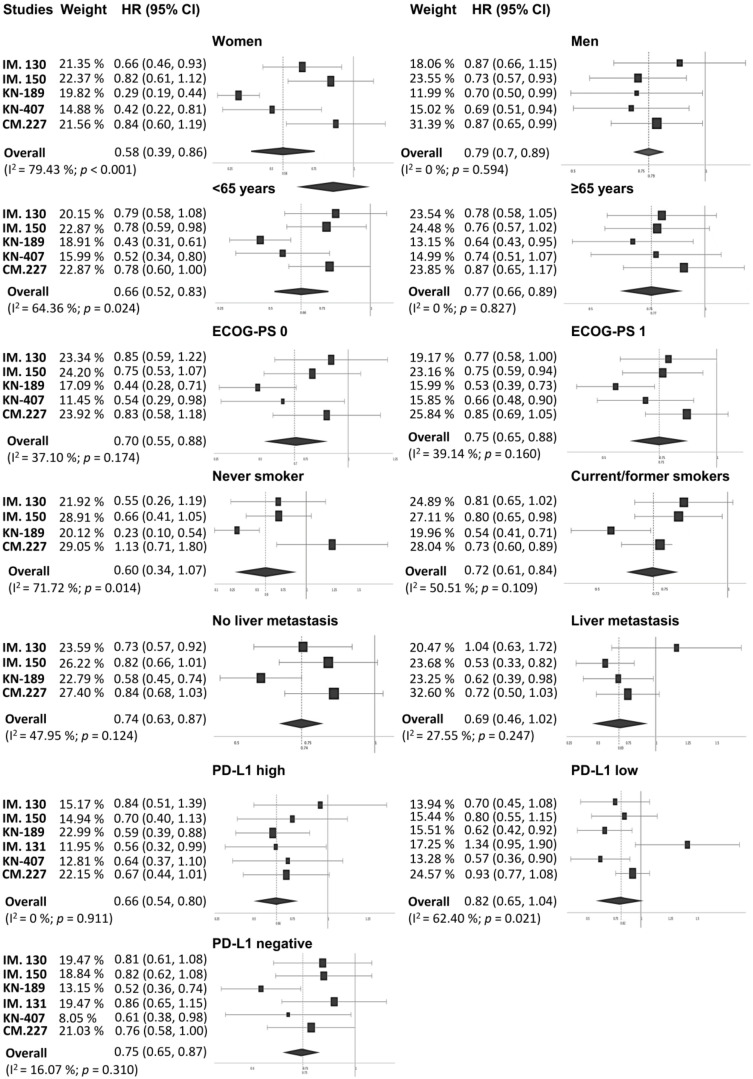
Forest plot of hazard ratios for overall survival (OS) in the different patient subgroups. CI, confidence interval; HR, hazard ratio; ECOG-PS, Eastern Cooperative Oncology Group performance status; IM., IMpower; KN, KEYNOTE; CM., CheckMate.

Regarding patients with liver metastasis, a specific benefit with atezolizumab plus bevacizumab was observed both in terms of OS and PFS. Further details on the OS and PFS subgroup analyses are shown in Figure 4 and Figure 5, respectively. Additional subgroup analyses based on the histology are available only for PFS and OS in Supplementary Figure S2.

## 4. Discussion

The optimal treatment strategy for advanced NSCLC has been the focus of several randomized clinical trials. Promising immunotherapy results in the second or later lines of therapy resulted in the approval of atezolizumab, pembrolizumab, and nivolumab [31,32,33,34,35]. Several clinical trials subsequently evaluated PD-(L)1 inhibitor–chemotherapy strategies in front-line treatment, some of which are included in this MA.

Our results demonstrate an overall benefit—both in terms of PFS and OS—of the addition of PD-(L)1 blockade. Although statistical significance was reached for the pooled HR for OS, substantial heterogeneity (I^2^ of 52.07%) across the seven trials was also identified. Furthermore, it is worth mentioning that the most recent data were considered for this MA in the vast majority of cases and that, to date, this is the first analysis to include results from CheckMate-227 part 2 [21]. Positive efficacy results have also been reported by Tun et al. [36], who included almost the same trials as those analyzed in this study (CheckMate-227 data were collected from part 1 [37]). Other meta-analyses have also reported improvements in the efficacy of the combined strategy. Differences in these may be explained by the trials included therein, such as the study by Chen et al., in which comparisons of immune checkpoint inhibitors against chemotherapy were also considered [38]; the study by Shen et al. [39] with broader inclusion criteria (e.g., studies that directly or indirectly investigated the ORR, the disease control response (DCR), or some safety endpoints); or the meta-analysis by Addeo et al. [40], in which studies using avelumab and durvalumab were also considered. Thus, our results support the evidence that a combination strategy of PD-(L)1 inhibitor plus chemotherapy may be beneficial compared to chemotherapy alone. Indeed, to date, the combination of pembrolizumab or atezolizumab with platinum-based chemotherapy, with or without bevacizumab, are EMA-approved options available for first-line treatment of advanced/metastatic NSCLC wildtype tumors.

With respect to subgroup analyses, overall benefits were reported across the different categories. Specifically, analysis in terms of PD-L1 marker yielded a statistically significant improvement in PFS regardless of the level of PD-L1 expression. In the case of OS, improvements were observed in patients with high PD-L1 expression and patients negative for this biomarker, but not in those with low levels, probably because of the moderate–high heterogeneity recorded in the pooled analysis (I^2^ = 67.01%; *p* = 0.016). It is also important to note that the studies included utilized different PD-L1 assay methods, possibly representing an additional confounding factor to be considered. Other subgroup analyses also resulted in important outcomes. Thus, this meta-analysis demonstrated that patients benefited from additional immunotherapy regardless of their age. It should be noted that the impact of advanced age on the effectiveness of immune checkpoint inhibitors has not been strongly established so far, highlighting the importance of these findings. Interestingly, combinations with pembrolizumab yielded the lowest HR values in terms of both PFS and OS in several subgroups, including women, patients <65 years, and patients with ECOG-PS = 0, pointing to a potential benefit in these individuals. With respect to liver metastasis, in the IMpower150 trial [23,24], improvements were reported both in terms of PFS and OS, suggesting a specific benefit with the atezolizumab and bevacizumab combination. Indeed, although other atezolizumab trials previously reported outcomes in patients with liver metastases, data from IMpower130 [41] and IMpower132 [42] showed no survival benefit with atezolizumab plus chemotherapy, supporting the benefits of adding the antiangiogenic agent in the combination [23]. Despite the fact that the updated KEYNOTE-189 analysis showed a clinical benefit of pembrolizumab-containing regimens over chemotherapy alone in patients with liver metastases (median OS 12.6 vs. 6.6, OS HR 0.62, 12-month OS rate 51% vs. 3%) [43], this baseline characteristic, in contrast with the IMpower trials, was not a stratification factor in the study.

Most clinical trials do not include advanced NSCLC patients with driver mutations. IMpower150 was the only study to include this type of patient, showing a positive trend in OS probably due to the addition of bevacizumab to the combination strategy, as previously discussed [43]. However, this therapeutic strategy for patients with EGFR/ALK mutations should be further confirmed in prospective, randomized studies.

This meta-analysis also has some limitations. First, as mentioned, the PD-L1 assay methods were not consistent across different studies. Thus, while PD-L1 immunohistochemistry was read on both tumor cells and tumor-infiltrating cells in the atezolizumab studies (IMpower) [20,24,25,29,43], PD-L1 expression was only measured on tumor cells in the trials assessing pembrolizumab (KEYNOTE) and nivolumab (CheckMate-227), [17,21,22,28]. Second, six of the included trials only provided interim analysis of the OS [17,20,22,24,25,28,29,43], which may misrepresent overall efficacy. Finally, the subgroup analysis was limited by the available information (PFS subgroup analyses were not assessed in CheckMate-227), and consequently caution must be exercised when interpreting the results. In this regard, certain limitations were also found with the available data of three of the studies, IMpower131, IMpower132, and CheckMate-227 part 2, whose results have only been published as congress abstracts and personal communications to date [20,21,29]. Despite these limitations, our results confirm those obtained in individual studies and are in line with the outcomes obtained in similar meta-analyses.

In conclusion, treatment with PD-(L)1 inhibitors resulted in significantly longer OS and PFS in stage IV NSCLC patients compared with chemotherapy alone. As a result, immunotherapy–chemotherapy combinations may be considered as a first-line strategy for these patients.

## Figures and Tables

**Table 1:** Characteristics and main outcomes of the studies included in the meta-analysis.

Study	Histology Expression	PD-L1 Expression	Primary Endpoint	Experimental Arm	Control Arm	Analysis Timing
IMpower130 [25]	Nonsquamous	All	PFS (ITT-WT *) OS (ITT-WT *)	Atezolizumab + (carboplatin + nab-paclitaxel) (*n* = 451)	Carboplatin + nab-paclitaxel (*n* = 228)	PFS: Final OS: Interim
IMpower150 [23,24]	Nonsquamous	All	PFS (ITT-WT *) OS (ITT-WT *)	Atezolizumab + (carboplatin + paclitaxel + bevacizumab) (*n* = 356)	Carboplatin + paclitaxel + bevacizumab (*n* = 336)	PFS: Final OS: Interim
KEYNOTE-189 [17,28]	Nonsquamous	All	PFS (ITT) OS (ITT)	Pembrolizumab + (carboplatin or cisplatin + pemetrexed) (*n* = 410)	Carboplatin or cisplatin + pemetrexed (*n* = 206)	PFS: Final OS: Interim
IMpower132 [20]	Nonsquamous	All	PFS (ITT) OS (ITT)	Atezolizumab + (carboplatin or cisplatin + pemetrexed) (*n* = 292)	Carboplatin or cisplatin + pemetrexed (*n* = 286)	PFS: Final OS: Interim
IMpower131 [29]	Squamous	All	PFS (ITT) OS (ITT)	Atezolizumab + (carboplatin + nab-paclitaxel) (*n* = 343)	Carboplatin + nab-paclitaxel (*n* = 340)	PFS: Final OS: Interim
KEYNOTE-407 [22]	Squamous	All	PFS (ITT) OS (ITT)	Pembrolizumab + (carboplatin + paclitaxel or nab-paclitaxel) (*n* = 278)	Carboplatin + paclitaxel or nab-paclitaxel (*n* = 281)	PFS: Final OS: Interim

* Patients with epidermal growth factor receptor (EGFR) or anaplastic lymphoma kinase (ALK) mutations excluded. PD-L1, programmed cell death-ligand 1; PFS, progression-free survival; OS, overall survival; ITT, intention-to-treat; WT, wildtype; +, plus (combination therapy).

## Supplementary Materials

The following are available online at https://www.mdpi.com/2077-0383/9/7/2093/s1: Figure S1: Forest plot of pooled odds ratios for overall response rate (ORR) in patients who received PD-(L)1 inhibitors plus chemotherapy vs. chemotherapy alone; Figure S2: Forest plot of pooled hazard ratios for (**A**) progression-free survival (PFS) and (**B**) overall survival in patients with nonsquamous or squamous NSCLC who received PD-(L)1 inhibitors plus chemotherapy vs. chemotherapy alone; Table S1: Treatments previously administered for nonmetastatic disease (data not available for atezolizumab studies); Table S2: Mutation status of IMpower150 [25,26] study patients; and Table S3: Characteristics of the patient population of the studies included in the meta-analysis.
