# Supplementary material for: PD-(L)1 Inhibitors in Combination with Chemotherapy as First-Line Treatment for Non-Small-Cell Lung Cancer: A Pairwise Meta-Analysis
Source: J Clin Med. 2020 Jul 3;9(7):2093. doi: 10.3390/jcm9072093 (PMC7408884; doi:10.3390/jcm9072093)
Supplement: Supplementary file 1 [file jcm-09-02093-s001.pdf]

Supplementary Material

Supplementary Figure S1. Forest plot of pooled odds ratios for overall response rate (ORR) in patients who received PD-(L)1 inhibitors plus chemotherapy *vs*. chemotherapy alone. **Treat.**, events in treatment arm/total; **Ctr.**, events in control arm/total; **CI**, confidence interval; **OR**, odds ratio.

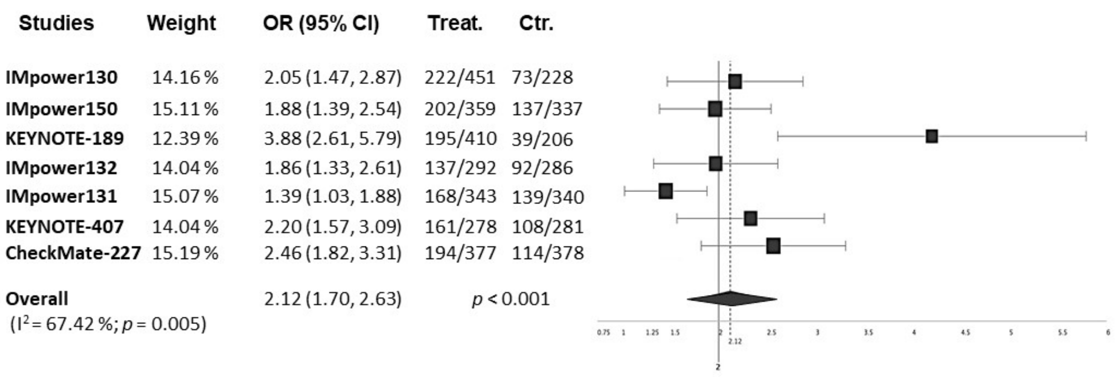

Supplementary Figure S2. Forest plot of pooled hazard ratios for (A) progression-free survival (PFS) and (B) overall survival in patients with non-squamous or squamous NSCLC who received PD-(L)1 inhibitors plus chemotherapy *vs*. chemotherapy alone. **HR**, hazard ratio; **CI**, confidence interval. **IM.**, IMpower; **KN.**, KEYNOTE; **CM**. CheckMate

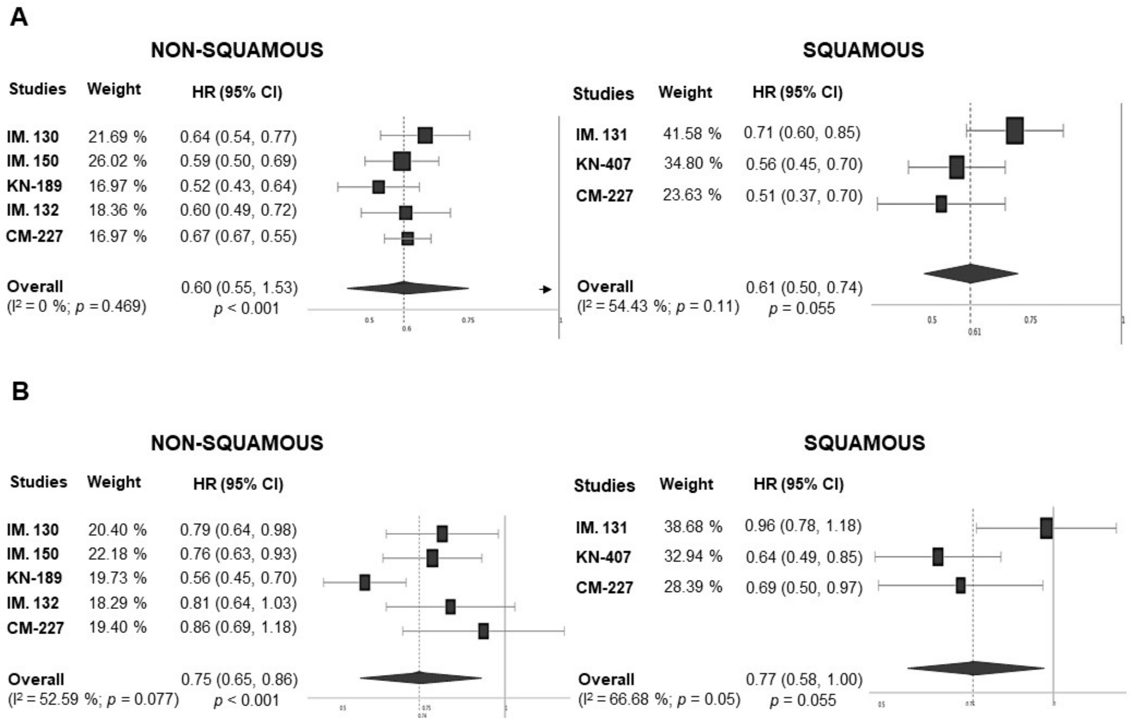

Supplementary Table S1. Treatments previously administered for nonmetastatic disease (data not available for atezolizumab studies)

**Studies**	**Thoracic radiation**		**Neoadjuvant therapy**		**Adjuvant therapy**	
	Control *n* (%)	Treatment *n* (%)	Control *n* (%)	Treatment *n* (%)	Control *n* (%)	Treatment *n* (%)
KEYNOTE-189 [30, 31]	20 (9.70)	28 (6.8)	6 (2.90)	5 (1.20)	14 (6.80)	25 (6.10)
KEYNOTE-407 [24]	22 (7.80)	17 (6.10)	8 (2.80)	5 (1.80)	NA	NA

NA, not applicable

Supplementary Table S2. Mutation status of IMpower150 [25, 26] study patients.

**Mutation status**	**Negative**		**Positive**	
	**Control arm** *n* **(%)**	**Experimental arm** *n* **(%)**	**Control arm** *n* **(%)**	**Experimental arm** *n* **(%)**
**EGFR**	345 (86.30)	352 (88)	45 (11.30)	34 (8.80)
**EML4-ALK**	375 (93.80)	383 (95.80)	21 (5.20)	13 (3.20)
**KRAS**	77 (19.20)	59 (14.80)	38 (9.50)	47 (11.80)

Supplementary Table S3. Characteristics of the patient population of the studies included in the meta-analysis.

Study	Experimental arm	Total *n*	Men *n* (%)	Median age (range)	Current or former smokers *n* (%)	PD-L1 high/low-intermediate/negative (%)	ECOG-PS 0/1/2 (%)	Liver metastasis *n* (%)	Bone metastasis *n* (%)	Brain metastasis *n* (%)
IMpower 130 [27]	Atezolizumab + [carbo + nab-paclitaxel]	451	266 (59)	64 [18-86]	403 (89)	20/28/52	42/58/0	69 (15)	126 (28)	NA
IMpower 150 [25, 26]	Atezolizumab + [carbo + paclitaxel + bevacizumab]	356	240 (60)	63 [31-89]	318 (79.50)	19/87/48	40.10/59.90/0	53 (13.20)	NA	NA
KEYNOTE-189 [30, 31]	Pembrolizumab + [carbo or cisplatin + pemetrexed]	410	254 (62)	65 [34-84]	362 (88.30)	32.2/31/31	45.40/53.90/0.2	66 (16)	NA	73 (17.80)
IMpower 132 [22]	Atezolizumab + [carbo or cisplatin + pemetrexed]	292	192 (65.8)	64 [31-85]	255 (87.30)	14.20/35.8/50	43.20/53.8/0	37 (12.70)	NA	NA
IMpower 131 [32]	Atezolizumab + [carbo + nab-paclitaxel]	343	279 (81)	65 [23-83]	311 (91)	15/38/47	34/66/0	70 (20)	NA	NA
KEYNOTE-407 [24]	Pembrolizumab + [carbo + paclitaxel or nab-paclitaxel]	278	220 (79.1)	65 [29-87]	256 (92.10)	26.30/37.10/34.20	26.30/76.70	NA	NA	20 (7.20)
CheckMate-227 [23]	Nivolumab and ipilimumab + Platinum-doublet chemotherapy* (*n* = 377)	377	264 (70)	63 [27-84]	317 (84)	24/34/40	35/64/1	87 (23)	109 (29)	NA

**Carboplatin or cisplatin plus gemcitabine or pemetrexed or paclitaxel

PD-L1, programmed cell death-ligand 1; **ECOG-PS**, Eastern Cooperative Oncology Group performance status; **Carbo**, carboplatin; **NA**, not applicable
